# Pancreatic head resection for carcinoma of the ampulla vateri – better long-term prognosis, but more postoperative complications

**DOI:** 10.1007/s00423-024-03319-7

**Published:** 2024-04-17

**Authors:** Simon Kuesters, Johanna Sundheimer, Uwe A. Wittel, Sophia Chikhladze, Stefan Fichtner-Feigl, Esther A. Biesel

**Affiliations:** 1 Department of General- and Visceral Surgery, University of Freiburg Medical Center, Faculty of Medicine, Freiburg, Germany; 2 Current address: Clinic for General-, Visceral- and Vascular Surgery, Fürst-Stirum-Klinik, Bruchsal, Germany

**Keywords:** Pancreatic cancer - ampullary carcinoma, Pancreatoduodenectomy, Postoperative complications, Oncological outcome

## Abstract

**Background:**

Pancreatoduodenectomies are complex surgical procedures with a considerable morbidity and mortality even in high-volume centers. However, postoperative morbidity and long-term oncological outcome are not only affected by the surgical procedure itself, but also by the underlying disease. The aim of our study is an analysis of pancreatoduodenectomies for patients with pancreatic ductal adenocarcinoma (PDAC) and ampullary carcinoma (CAMP) concerning postoperative complications and long-term outcome in a tertiary hospital in Germany.

**Methods:**

The perioperative and oncological outcome of 109 pancreatic head resections performed for carcinoma of the ampulla vateri was compared to the outcome of 518 pancreatic head resections for pancreatic ductal adenocarcinoma over a 20 year-period from January 2002 until December 2021. All operative procedures were performed at the University Hospital Freiburg, Germany. Patient data was analyzed retrospectively, using a prospectively maintained SPSS database. Propensity score matching was performed to adjust for differences in surgical and reconstruction technique. Primary outcome of our study was long-term overall survival, secondary outcomes were postoperative complications and 30-day postoperative mortality. Postoperative complications like pancreatic fistula (POPF), postpancreatectomy hemorrhage (PPH) and delayed gastric emptying (DGE) were graded following current international definitions. Survival was estimated using Kaplan Meier curves and log-rank tests. A *p*-value < 0.05 was considered statistically significant.

**Results:**

Operation time was significantly longer in PDAC patients (432 vs. 391 min, *p* < 0.001). The rate of portal vein resections was significantly higher in PDAC patients (*p* < 0.001). In CAMP patients, a pancreatogastrostomy as reconstruction technique was performed more frequently compared to PDAC patients (48.6% vs. 29.9%, *p* < 0.001) and there was a trend towards more laparoscopic surgeries in CAMP patients (*p* = 0.051). After propensity score matching, we found no difference in DGE B/C and PPH B/C (*p* = 0.389; *p* = 0.517), but a significantly higher rate of clinically relevant pancreatic fistula (CR-POPF) in patients with pancreatoduodenectomies due to ampullary carcinoma (30.7% vs. 16.8%, *p* < 0.001). Long-term survival was significantly better in CAMP patients (42 vs. 24 months, *p* = 0.003).

**Conclusion:**

Patients with pancreatoduodenectomies due to ampullary carcinomas showed a better long-term oncological survival, by reason of the better prognosis of this tumor entity. However, these patients often needed a more elaborated postoperative treatment due to the higher rate of clinically relevant pancreatic fistula in this group.

**Supplementary Information:**

The online version contains supplementary material available at 10.1007/s00423-024-03319-7.

## Introduction

Pancreatic cancer is – despite efforts in research and clinical treatment over the last decades – still a malignancy with a high mortality and increasing incidence rates during recent years [1]. In spite of slight improvements of 5-year overall survival from < 5% in the 1990s to up to 9% in the USA and Europe in 2019 [2, 3], the survival rates for pancreatic cancer remain low [4]. On the other hand, there are ampullary carcinomas, also known as carcinomas of the ampulla of Vater, which represent with only 0.2% of all gastrointestinal tumors and approximately 10% of the periampullary carcinomas a rather rare tumor entity [5–7]. Ampullary carcinomas tend to present earlier than the pancreatic adenocarcinoma [8] and seem to be less biological aggressive, leading to a better overall survival than other types of periampullary carcinomas [8]. Due to its localization, the majority of ampullary carcinomas present with jaundice and other symptoms similar to distal cholangiocarcinomas or pancreatic head carcinoma like diarrhea, steathorea and gastrointestinal bleeding, but the symptoms occur regularly earlier than in pancreatic cancer patients [5]. Early lymph node metastasis is common [9] and pancreatoduodenectomy still is the treatment of choice for ampullary adenocarcinomas [9]. However, pancreatoduodenectomies still are complex surgical procedures with a considerable rate of postoperative morbidity and mortality, even in high-volume centers [10–12]. The most common causes of postoperative morbidity following pancreatoduodenectomies are pancreatic fistulas (POPF), postpancreatectomy hemorrhage (PPH) and delayed gastric emptying (DGE) [13]. In most cases, conservative treatment or interventional procedures have proven a high success rate in management of complications and therefore are the treatment of choice, however, revision surgery is necessary in 10 - 20% of cases [14, 15]. Aim of our study was the comparison of pancreatoduodenectomy for ampullary cancer with pancreatoduodenectomy for pancreatic ductal adenocarcinomas in a high-volume center with regard to postoperative complications and mortality, the need of surgical revisions and long-term outcomes of patients.

## Materials and methods

### Patient collective

**Fig. 1:**
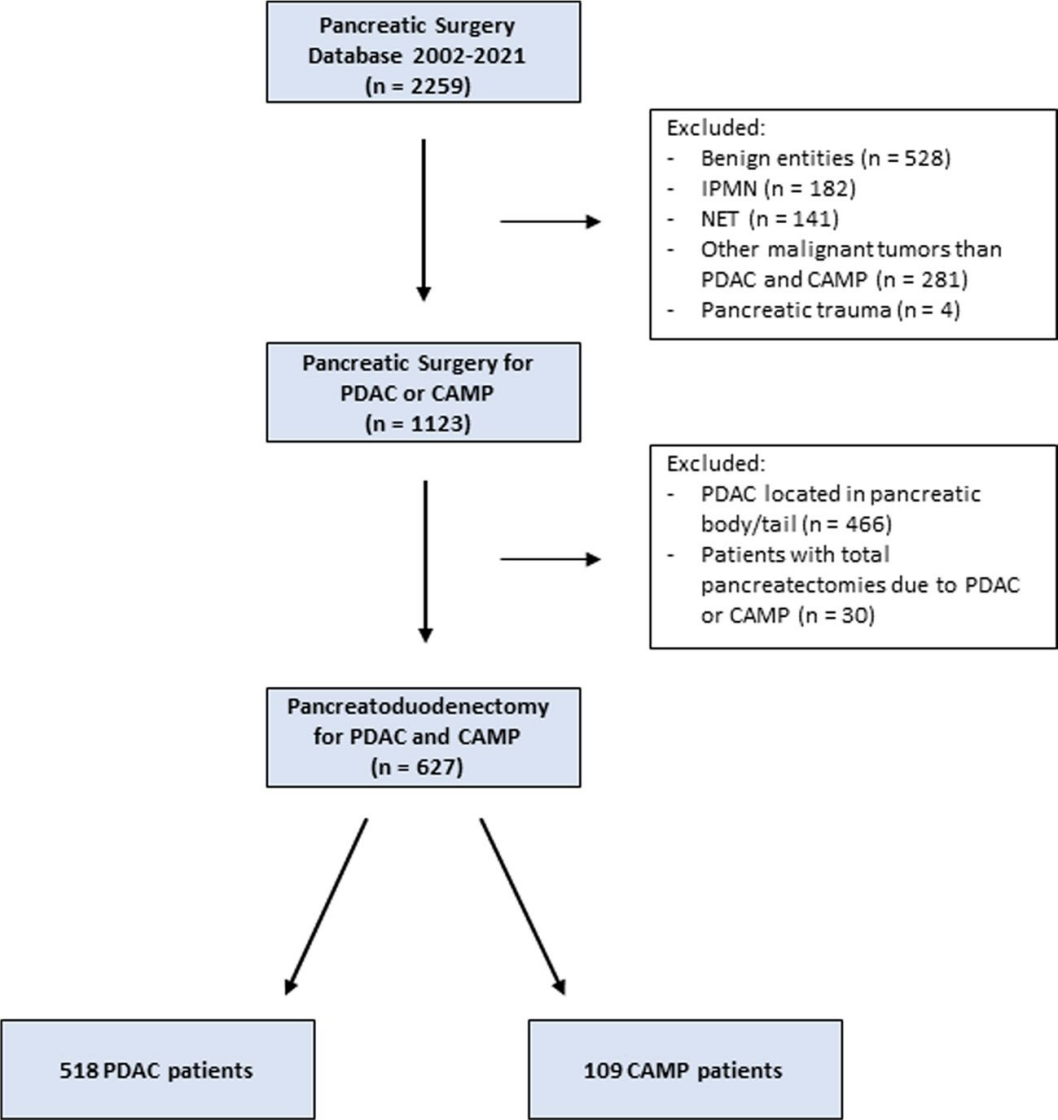
Flow Chart of patient collective. PDAC = pancreatic ductal adenocarcinoma. CAMP = ampullary carcinoma. NET = neuroendocrine tumor. IPMN = intraductal papillary mucinous neoplasm

Our study was performed as a single center study at the University Medical Center Freiburg. Clinical data of 627 patients with pancreatoduodenectomies either due to pancreatic ductal adenocarcinoma (PDAC) or due to ampullary carcinoma (CAMP) in our institution between January 2002 and December 2021 were evaluated retrospectively, using a prospectively maintained pancreatic surgery database. Patients with total pancreatectomies were excluded due to reasons of homogenization. Details concerning patient collective are shown in Fig. 1.

### Data collection and variables

Data collection at our clinic is performed continuously using a prospectively maintained pancreatic surgery SPSS database. Besides demographic data, preoperative BMI and ASA-score, variables include details on duration of surgery, blood transfusions and surgical techniques as well as duration of hospital stay and in-hospital-mortality. Follow-up studies with general practitioners or oncologists and cancer registries provide information on long-term survival. Primary outcome of our study was long-term overall survival, secondary outcomes were postoperative complications and 30-day postoperative mortality. Postoperative complications such as postpancreatectomy hemorrhage (PPH), pancreatic fistula (POPF) or delayed gastric emptying (DGE) were graded using current international definitions of the International Study Group on Pancreatic Surgery (ISGPS) [16–19].

### Statistical analysis

Statistical analysis was performed using SPSS (IBM SPSS Statistics for Windows, Version 28.0. IBM Corp., Armonk, NY, USA) and GraphPad Prism (GraphPad Software, Version 10, San Diego, CA, USA). After performing explorative analysis and descriptive statistics, statistical significance was examined by using chi-square tests and Fisher´s exact tests for categorical variables and ANOVA for continuous variables. Survival status was obtained from the comprehensive cancer center registry at our institution and/or from the computerized hospital information system. Overall survival was analyzed using the Kaplan–Meier method, differences in overall survival were assessed using log-rank tests and uni- and multivariable Cox regression models (forward selection method with likelihood ratio). Results with a *p*-value < 0.05 were considered statistically significant. Propensity score matching was performed to reduce bias for different surgical techniques. Multivariable logistic regression model was performed to generate the propensity score. The following factors were included in this model: laparoscopic resection, technique of reconstruction (pancreaticojejuno- vs. pancreatogastrostomy) and portal vein resection. After establishing the propensity score, 1:1 matching using the nearest-neighbour matching was performed with a caliper of 0.01 without replacement. Post hoc balance diagnostic was performed using mean standardized differences [20].

### Ethics

Data collection and analysis were performed in accordance with the Declaration of Helsinki and were approved by the local ethics committee (Ethics Committee of Albert-Ludwigs-University Freiburg, Germany, EK-No. 23-1424-S1-retro).

## Results

### Patient characteristics and intraoperative parameters

The total number of patients included in this study was 627 (109 (17.4 %) in the CAMP- and 518 (82.6 %) in the PDAC-group). In the unmatched cohort, we found no difference concerning sex (51.9% vs. 54.1% male patients, *p* = 0.676) or age of patients (66.3 vs. 66.5 years, *p* = 0.848). Mean body mass index (BMI) was 25.1 kg/m^2^ in PDAC patients vs. 25.0 kg/m^2^ in CAMP patients (*p* = 0.775). There was no significant difference in ASA (American Society of Anesthesiology) score, most patients in both groups had an ASA-score of 2 or 3 (93.3 % PDAC vs 95.4% CAMP, *p* = 0.680). There was no difference in relevant comorbidities concerning coronary heart disease, hypertension, lung disease, liver disease or renal insufficiency. However, there was a trend towards more preoperative diabetes mellitus in the pancreatic adenocarcinoma group (24.2% vs 15.7%, *p* = 0.057). Concerning preoperative bile duct stenting, we found no difference between both groups (PDAC 55.3% vs. CAMP 63.6%, *p* = 0.116). Significantly more patients in the PDAC group received neoadiuvant (7.7% vs. 0.0%, *p* = 0.005) or adiuvant chemotherapy (55.2 vs. 25.6%, *p* < 0.001), respectively. Operation time for CAMP was significantly shorter than in the PDAC-group (mean operative time 391 min vs. 432 min, *p* < 0.001). Venous resections were necessary in 42.3% of PDAC-patients but only in 8 patients (7.4%) in the CAMP-group (*p* < 0.001). Intraoperative assessment of the pancreatic texture revealed a soft pancreas in 70.6% of CAMP, but only in 38.1% of PDAC-cases (*p* < 0.001). Surgeons were free to choose a suitable reconstruction method according to the intraoperative situation. Reconstruction techniques in the CAMP group were pancreatogastrostomy in 48.6% (*n* = 53) of patients and pancreaticojejunostomy in 51.4% (*n* = 56). There was a significantly different distribution in the PDAC-group: pancreatogastrostomy was performed in 29.9% (*n* = 155) and pancreaticojejunostomy in 70.1% (*n* = 363) of patients. There was a trend towards more laparoscopically assisted resections in the CAMP group compared with the PDAC group (PDAC 21.6%, *n* = 112; CAMP 30.3%, *n* = 33; *p* = 0.051). Patient characteristics and intraoperative parameters of the unmatched cohort are summarized in Table 1.

**Table 1:** Demographic and surgical parameters of the unmatched cohort

	PDAC (*n* = 518)	CAMP (*n* = 109)	*p* value	Mean standardized difference
Demographic parameters and comorbidities				
Age, years	66.3 (11.1)	66.5 (11.3)	0.848	0.018
Sex				
- male	269 (51.9)	59 (54.1)	0.676	0.044
- female	249 (48.1)	50 (45.9)		
BMI, kg/m2	25.1 (4.5)	25.0 (3.8)	0.775	0.024
ASA classification				
- ASA 1	22 (4.2)	4 (3.7)	0.783	0.040
- ASA 2	249 (48.1)	57 (52.3)	0.423	0.084
- ASA 3	234 (45.2)	47 (43.1)	0.695	0.042
- ASA 4	13 (2.5)	1 (0.9)	0.306	0.124
Comorbidities (*n* = 513)	343 (79.0)	65 (82.3)	0.511	0.083
Coronary heart disease (*n* = 508)	61 (14.2)	11 (14.1)	0.984	0.002
Hypertension (*n* = 510)	235 (54.4)	45 (57.7)	0.590	0.066
Lung disease (*n* = 513)	73 (16.8)	17 (21.5)	0.313	0.120
Renal disease (*n* = 513)	43 (9.7)	7 (8.4)	0.712	0.045
Liver disease (*n* = 503)	122 (28.6)	18 (23.7)	0.381	0.112
Diabetes mellitus	125 (24.2)	17 (15.7)	0.057	0.214
Alcohol abuse	47 (9.8)	10 (10.0)	0.939	0.007
Nicotin abuse	97 (20.2)	23 (23.0)	0.524	0.068
Preoperative icterus	343 (67.0)	70 (65.4)	0.754	0.034
Preoperative bile duct stent	283 (55.3)	68 (63.6)	0.116	0.170
Neoadiuvant chemotherapy (*n* = 613)	40 (7.7)	0 (0.0)	**0.005**	0.408
Adiuvant chemotherapy (*n* = 543)	250 (55.2)	23 (25.6)	**<0.001**	0.708
Surgical parameters				
Duration of surgery, minutes	432 (95.3)	391 (86.4)	**0.001**	0.455
Venous resection	219 (42.3)	8 (7.4)	**< 0.001**	0.883
Soft pancreas (*n* = 303)	96 (38.1)	36 (70.6)	**< 0.001**	0.395
Laparoscopical-assisted resection	112 (21.6)	33 (30.3)	0.051	0.199
Reconstruction technique				
- pancreatogastrostomy	155 (29.9)	53 (48.6)	**< 0.001**	0.390
- pancreaticojejunostomy	363 (70.1)	56 (51.4)	**< 0.001**	0.390
Diameter of pancreatic main duct, mm (*n* = 118)	5.1 (2.2)	3.8 (1.7)	**0.038**	0.661

Data are presented as n (%), or mean +/- SD. *SD* = standard deviation, *PDAC* pancreatic ductal adenocarcinoma, *CAMP* ampullary carcinoma, *BMI* body mass index, *ASA* American Society of Anesthesiologists. *p*-values < 0.05 are emphasised in bold print

### Histopathological results

In the unmatched cohort, tumor-free resection margins were achieved in 97.2% of cases in CAMP patients and in 75.8% of patients with PDAC (*p* < 0.001). Most PDAC tumors were of T3 state (71.3%) whereas in the CAMP group, there were nearly as many T2 as T3 tumors (34.3% and 39.8%). Remarkably, there were more T4 tumors in the CAMP group than in the PDAC group (12.0% vs. 2.3%). Concerning lymph node affection, there was also a significant difference with a higher N0-rate in the CAMP-group (46.8% vs. 29.3%, *p* < 0.001). Histopathological results of the unmatched cohort are shown in Table 2.

**Table 2:** Histopathological results of the unmatched cohort

	PDAC (*n* = 518)	CAMP (*n* = 109)	*p* value
Grading (*n* = 600)			
- G1	14 (2.8)	8 (7.6)	**0.018**
- G2	281 (56.8)	61 (58.1)	0.803
- G3	194 (39.2)	36 (34.3)	0.348
- G4	6 (1.2)	0 (0.0)	0.257
Resection margin (*n* = 626)			
- R0	392 (75.8)	106 (97.2)	**< 0.001**
- R1	117 (22.6)	3 (2.8)	**< 0.001**
- R2	8 (1.5)	0 (0.0)	0.191
TNM classification (*n* = 623)			
- T1	32 (6.2)	15 (13.9)	**0.006**
- T2	104 (20.2)	37 (34.3)	**0.001**
- T3	367 (71.3)	43 (39.8)	**< 0.001**
- T4	12 (2.3)	13 (12.0)	**< 0.001**
- N0	151 (29.3)	51 (46.8)	**< 0.001**
- N1	315 (61.2)	53 (48.6)	**0.016**
- N2	49 (9.5)	5 (4.6)	0.096

Data are presented as *n* (%). *PDAC* pancreatic ductal adenocarcinoma, *CAMP* ampullary carcinoma. *p*-values < 0.05 are emphasised in bold print

### Postoperative outcome: Complications and survival in patients with CAMP compared to PDAC patients in the unmatched and matched cohort

In the unmatched cohort, there was no significant difference in overall postoperative complication rate between CAMP and PDAC patients (64.2% vs. 56.1%, *p* = 0.119), but there were significantly more surgical complications in the CAMP-group than in the PDAC-group (57.9% vs. 40.9%, *p* = 0.001), mainly caused by a significantly higher rate of clinically relevant pancreatic fistula (CR-POPF) with 30.5% CR-POPF in CAMP patients compared to only 12.4% in PDAC patients (*p* < 0.001). Concerning delayed gastric emptying and postpancreatectomy hemorrhage, we found no difference between both groups (DGE 26.2% CAMP vs. 22.8% PDAC, *p* = 0.455; PPH 6.6% CAMP vs. 8.6% PDAC, *p* = 0.534). More patients with ampullary carcinomas needed a conservative therapy following pancreatoduodenectomy (73.1% vs. 58.6%, *p* = 0.006). Interestingly, the rate of acute kidney failure was significantly higher in PDAC patients (4.1% vs. 0.0%, *p* = 0.034). There was no difference concerning the need of surgical revisions between both groups (12.8% PDAC vs. 11.9% CAMP, *p* = 0.810). Postoperative 30-day-mortality was similar in both groups (4.1% vs. 3.7%, *p* = 0.849). The length of hospital stay was significantly longer in the CAMP group due to more surgical complications in the postoperative course (19 vs. 17 days, *p* = 0.012); however, there was no significant difference concerning the length of stay on the intensive care unit (ICU) (6 vs. 5 days, *p* = 0.562). Postoperative complications and survival of the unmatched cohort are summarized in Table 3.

**Table 3:** Postoperative complications and overall survival of the unmatched cohort

	PDAC (*n* = 518)	CAMP (*n* = 109)	*p* value
DGE B/C	117 (22.8)	28 (26.2)	0.455
PPH B/C	37 (8.6)	6 (6.6)	0.534
CR-POPF	64 (12.4)	33 (30.5)	**< 0.001**
Blood transfusion	97 (18.8)	12 (11.0)	0.052
Wound infection	67 (13.0)	21 (19.6)	0.071
Urinary tract infection	33 (6.4)	2 (1.9)	0.063
Thrombembolism	14 (2.7)	3 (2.8)	0.956
Intraabdominal abscess	55 (10.6)	13 (12.0)	0.671
Pneumonia	21 (4.1)	3 (2.8)	0.528
Reintubation	22 (4.3)	4 (3.7)	0.808
Sepsis	21 (4.1)	2 (1.9)	0.267
Acute kidney failure	21 (4.1)	0 (0.0)	**0.034**
Insufficiency pancreaticojejunostomy	21 (4.1)	4 (3.7)	0.849
Insufficiency pancreatogastrostomy	12 (2.3)	7 (6.4)	**0.023**
Insufficiency biliodigestive anastomosis	11 (2.1)	1 (0.9)	0.401
Postoperative mortality	21 (4.1)	4 (3.7)	0.849
Any complication	290 (56.1)	70 (64.2)	0.119
Surgery-related complication	212 (40.9)	62 (57.9)	**0.001**
Surgical revision	66 (12.8)	13 (11.9)	0.810
Postoperative interventional therapy (*n* = 624)	132 (25.6)	33 (30.6)	0.286
Postoperative conservative therapy	299 (58.6)	76 (73.1)	**0.006**
Hospital stay in days (median, range)	17 (2 – 329)	19 (6 – 377)	**0.012**
Intensive Care Unit in days (median, range)	5 (1 – 68)	6 (2 – 52)	0.562
Median overall survival, months (95% CI)	21 (18.9 – 23.1)	53 (19.2 -86.8)	**<0.001**

Data are presented as n (%), mean +/- SD or median (range). *SD* standard deviation, *PDAC* pancreatic ductal adenocarcinoma, *CAMP* ampullary carcinoma, *DGE* delayed gastric emptying, *PPH* postpancreatectomy hemorrhage, *CR-POPF* clinical relevant pancreatic fistula, *CI* confidence interval. *p*-values < 0.05 are emphasised in bold print

**Fig. 2:**
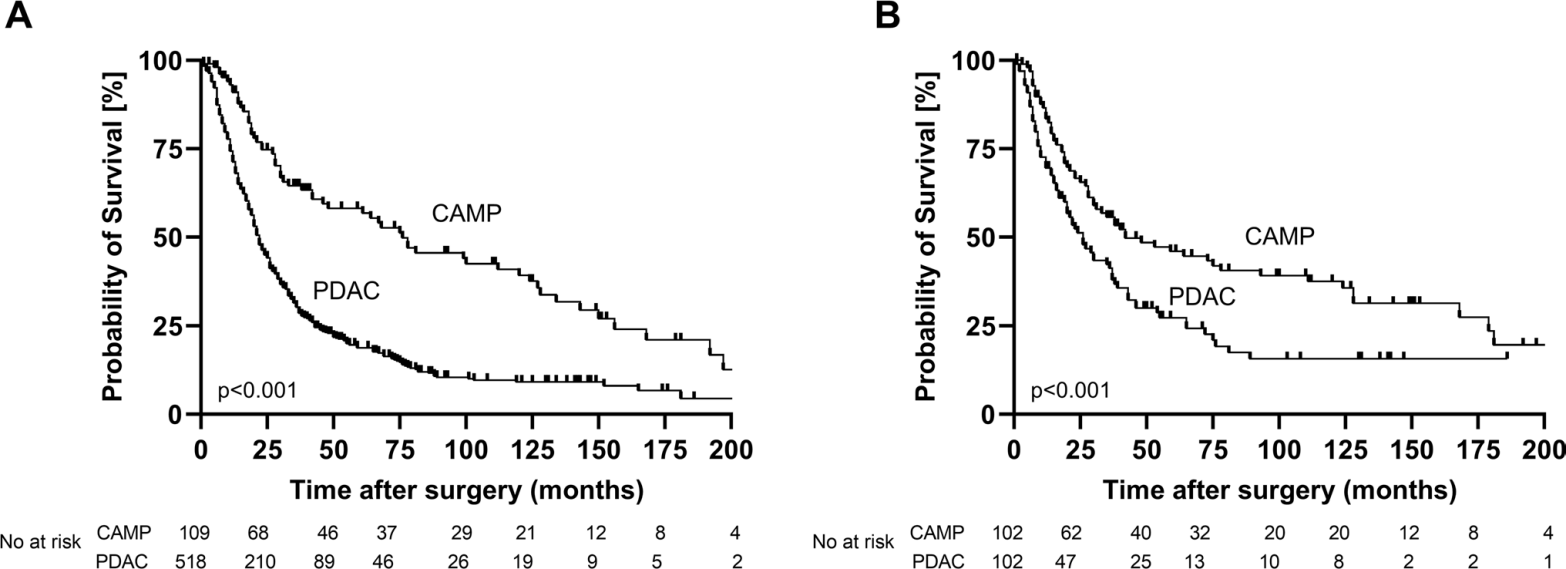
Median overall survival after pancreatoduodenectomy for ampullary and pancreatic carcinoma. **A**: Overall survival in the unmatched cohort. **B**: Overall survival after propensity score matching. PDAC = pancreatic ductal adenocarcinoma. CAMP = ampullary carcinoma

**Fig. 3:**
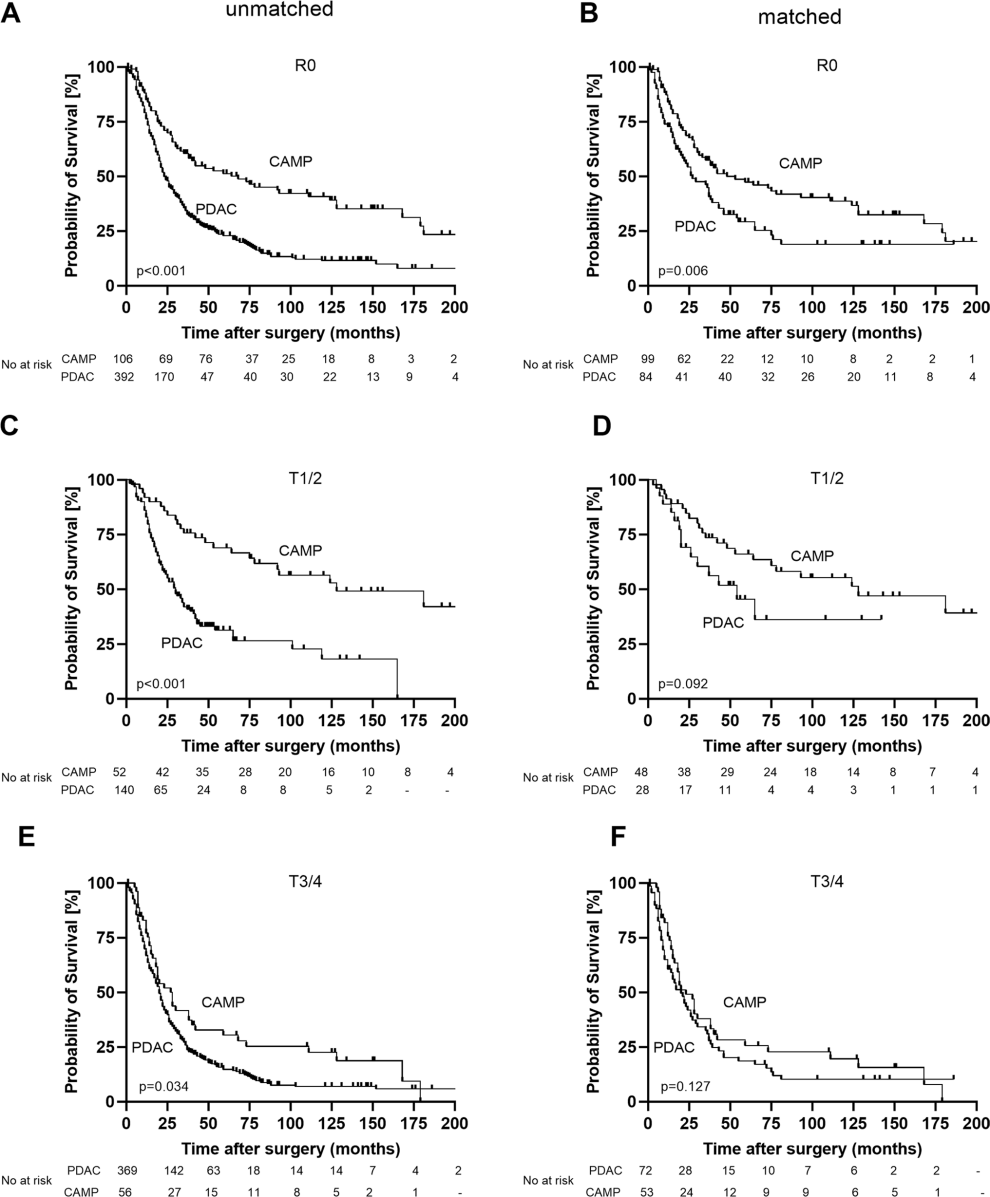
Median overall survival for R0-resections as well as for early and advanced primary tumor stages in the unmatched cohort and after propensity score matching. **A**: Overall survival of patients with R0-resections in the unmatched cohort. **B**: Overall survival of patients with R0-resections after propensity score matching. **C**: Overall survival of T1/T2 primary tumors in the unmatched cohort. **D**: Overall survival of T1/T2 primary tumors after propensity score matching. **E**: Overall survival of T3/T4 primary tumors in the unmatched cohort. **F**: Overall survival of T3/T4 primary tumors after propensity score matching. PDAC = pancreatic ductal adenocarcinoma. CAMP = ampullary carcinoma

Survival data were available for 516 PDAC- and 109 CAMP-patients. Median overall survival of CAMP patients (all T-, N- and R- states) was 53 months (95%-CI 19.2 - 86.8 months) compared to 21 months (95%-CI 18.9 – 23.1 months) in the PDAC group (*p* < 0.001) (Fig. 2A). Considering only tumors with R0 resection status, median survival in the CAMP group was 59 months (95%-CI 26.9 – 91.1 months; *n* = 106) vs. 23 months (95%-CI 20.4 – 25.6 months) in the PDAC group (*n* = 392; *p* < 0.001) (Fig. 3A), whereas survival after R1 resection was 14 months for CAMP patients (95%-CI 0.0 – 28.4 months; *n* = 3) and 13 months for PDAC patients (95%-CI 7.4 – 18.5 months; *n* = 116) (*p* = 0.285).

We performed a multivariable logistic regression model for development of the propensity score (details are shown in Supplementary Table 1). After 1:1 matching using the nearest-neighbour method, we identified 204 patients (102 PDAC patients and 102 CAMP patients) with comparable baseline and surgical characteristics (Table 4). Covariates which were used for development of the propensity score showed mean standardized differences <= 0.01 indicating adequate balance of the matched variables.

**Table 4:** Baseline characteristics and surgical parameters after propensity score matching

	PDAC (*n* = 102)	CAMP (*n* = 102)	*p* value	Mean standardized difference
Demographic parameters and comorbidities				
Age, years	64.5 (12.2)	66.5 (11.2)	0.200	0.170
Sex				
- male	53 (52.0)	58 (56.9)	0.482	0.098
- female	49 (48.0)	44 (43.1)		
BMI, kg/m2	25.5 (4.1)	24.9 (3.7)	0.292	0.154
ASA classification				
- ASA 1	8 (7.8)	4 (3.9)	0.234	0.167
- ASA 2	54 (52.9)	52 (51.0)	0.779	0.038
- ASA 3	39 (38.2)	45 (44.1)	0.393	0.120
- ASA 4	1 (1.0)	1 (1.0)	1.000	0.000
Comorbidities (*n* = 157)	64 (78.0)	61 (81.3)	0.610	0.082
Coronary heart disease (*n* = 153)	10 (12.7)	11 (14.9)	0.692	0.063
Hypertension (*n* = 154)	38 (47.5)	42 (56.8)	0.251	0.187
Lung disease (*n* = 155)	17 (21.3)	15 (20.0)	0.848	0.027
Renal disease (*n* = 157)	6 (7.6)	6 (7.6)	1.000	0.000
Liver disease (n = 150)	23 (29.5)	15 (20.8)	0.223	0.201
Diabetes mellitus	19 (18.8)	16 (15.7)	0.556	0.082
Alcohol abuse	11 (12.2)	10 (10.5)	0.716	0.053
Nicotin abuse	19 (21.1)	23 (24.2)	0.615	0.074
Preoperative icterus	70 (70.0)	66 (65.3)	0.481	0.101
Preoperative bile duct stent	59 (58.4)	65 (64.4)	0.386	0.123
Neoadiuvant chemotherapy (n = 191)	9 (8.8)	0 (0.0)	**0.004**	0.439
Adiuvant chemotherapy (n = 168)	48 (57.8)	23 (27.1)	**<0.001**	0.762
Surgical parameters				
Duration of surgery, minutes	425 (97.6)	393 (88.7)	**0.015**	0.343
Venous resection	8 (7.8)	8 (7.8)	1.000	0.000
Soft pancreas (n = 98)	27 (55.1)	34 (69.4)	0.345	0.170
Laparoscopical-assisted resection	31 (30.4)	31 (30.4)	1.000	0.000
Reconstruction technique				
- pancreatogastrostomy	48 (47.1)	48 (47.1)	1.000	0.000
- pancreaticojejunostomy	54 (52.9)	54 (52.9)	1.000	0.000
Diameter of pancreatic main duct, mm (n = 35)	5.2 (2.3)	3.8 (1.7)	0.077	0.692

Data are presented as n (%), or mean +/- SD. *SD* standard deviation, *PDAC* pancreatic ductal adenocarcinoma, *CAMP* ampullary carcinoma, *BMI* body mass index, *ASA* American Society of Anesthesiologists. *p*-values < 0.05 are emphasised in bold print

In the matched cohort, we found no significant differences concerning delayed gastric emptying (DGE B/C 19.0% PDAC vs. 24.0% CAMP, p = 0.389) and postpancreatectomy hemorrhage (PPH B/C 9.9% PDAC vs. 7.1% CAMP, *p* = 0.517) as well as concerning wound infections (19.8% vs. 18.0%, *p* = 0.744) or intraabdominal abscesses (12.9% vs. 11.9%, *p* = 0.831). Overall complications and 30-day-mortality were distributed equal as well between PDAC and CAMP patients (complications 56.4% vs. 64.7%, *p* = 0.228; mortality 4.0% vs. 3.9%, *p* = 0.989). There was just a trend concerning more postoperative surgical complications in CAMP patients (57.0% vs. 45.1 %, *p* = 0.091); however, patients with ampullary carcinomas still presented with a significantly higher rate of clinically relevant pancreatic fistula (CR-POPF 30.7% vs. 16.8% in PDAC patients, *p* < 0.001) and required significantly more conservative treatment following surgery (71.4% vs. 54.0%, *p* = 0.011). Details on histopathological results and postoperative complications in the matched cohort are summarized in Tables 5 and 6. Moreover, in the matched cohort, an improved overall survival of CAMP patients was consistent with 42 months median overall survival (95%-CI 17.1 - 66.9 months) in CAMP patients compared to 24 months (95%-CI 15.7 - 32.3 months) in PDAC patients (*p* = 0.003) (Fig. 2B). Considering only patients with R0 resections in the matched cohort, there was still a significantly better overall survival in patients with ampullary carcinomas (42 (95%-CI 15.1 – 68.9) vs. 26 (95%-CI 12.4 – 39.6) months, *p* = 0.006) (Fig. 3B). Moreover, by dividing the patients in two groups of either early or advanced primary tumors (T1/T2 vs. T3/T4), we found a significantly better overall survival for CAMP patients in the unmatched cohort (T1/T2 CAMP 128 (95%-CI 29.6 – 226.4) vs. PDAC 29 (95%-CI 21.3 – 36.7) months, *p* < 0.001; T3/T4 CAMP 27 (95%-CI 19.2 – 34.8) vs. PDAC 20 (95%-CI 18.0 – 22.0) months, *p* = 0.034) and still a trend towards a better overall survival for the early primary tumor stages of CAMP patients in the matched cohort (T1/T2 CAMP 128 (95%-CI 36.7 – 219.3) vs. PDAC 43 (95%-CI 10.9 – 75.1) months, *p* = 0.092; T3/T4 CAMP 20 (95%-CI 12.6 – 27.4) vs. PDAC 20 (95%-CI 12.1 – 27.9) months, *p* = 0.127). Survival curves for the different primary tumor stages in the unmatched and matched cohort are shown in Fig. 3C-F. Our analyses show that the prognosis of CAMP patients is altogether favorable in comparison to PDAC patients. In order to strengthen these data, we additionally performed Cox regression analyses in the unmatched cohort, highlighting the significant independent prognostic relevance of CAMP in comparison to PDAC. In these analyses, also the factors for propensity score matching were included. We performed uni- and multivariable Cox regression models using a forward selection method (forward variable selection, p(in) < 0.05, p(out) > 0.10, likelihood ratio). The results from this model can be found in Table 7.

**Table 5:** Histopathological results after propensity score matching

	PDAC (*n* = 102)	CAMP (*n* = 102)	*p* value
Grading (*n* = 195)			
- G1	3 (3.1)	6 (6.1)	0.313
- G2	59 (60.8)	59 (60.2)	0.929
- G3	33 (34.0)	33 (33.7)	0.959
- G4	2 (2.1)	0 (0.0)	0.153
Resection margin			
- R0	84 (82.4)	99 (97.1)	**<0.001**
- R1	15 (14.7)	3 (2.9)	**0.003**
- R2	3 (2.9)	0 (0.0)	0.081
TNM classification (*n* = 201)			
- T1	9 (9.0)	12 (11.9)	0.504
- T2	19 (19.0)	36 (35.6)	**0.008**
- T3	71 (71.0)	40 (39.6)	**<0.001**
- T4	1 (1.0)	13 (12.9)	**<0.001**
- N0	34 (34.0)	47 (46.1)	0.080
- N1	58 (58.0)	50 (49.0)	0.201
- N2	8 (8.0)	5 (4.9)	0.370

Data are presented as n (%). *PDAC* pancreatic ductal adenocarcinoma, *CAMP* ampullary carcinoma. *p*-values < 0.05 are emphasised in bold print

**Table 6:** Surgical complications and overall survival after propensity score matching

	PDAC (*n* = 102)	CAMP (*n* = 102)	*p* value
DGE B/C	19 (19.0)	24 (24.0)	0.389
PPH B/C	9 (9.9)	6 (7.1)	0.517
CR-POPF	16 (16.8)	31 (30.7)	**< 0.001**
Blood transfusion	14 (13.9)	9 (8.8)	0.258
Wound infection	20 (19.8)	18 (18.0)	0.744
Urinary tract infection	5 (5.0)	2 (1.0)	0.248
Thrombembolism	2 (2.0)	3 (3.0)	0.643
Intraabdominal abscess	13 (12.9)	12 (11.9)	0.831
Pneumonia	3 (3.0)	3 (3.0)	1.000
Reintubation	6 (5.9)	3 (3.0)	0.313
Sepsis	7 (6.9)	2 (2.0)	0.088
Acute kidney failure	2 (2.0)	0 (0.0)	0.157
Insufficiency pancreaticojejunostomy	8 (7.9)	4 (3.9)	0.227
Insufficiency pancreatogastrostomy	4 (4.0)	6 (5.9)	0.527
Insufficiency biliodigestive anastomosis	2 (2.0)	1 (1.0)	0.555
Postoperative mortality	4 (4.0)	4 (3.9)	0.989
Any complication	57 (56.4)	66 (64.7)	0.228
Surgery-related complication	46 (45.1)	57 (57.0)	0.091
Surgical revision	16 (15.8)	12 (11.8)	0.400
Postoperative interventional therapy (*n* = 201)	30 (30.0)	30 (29.7)	0.963
Postoperative conservative therapy	54 (54.0)	70 (71.4)	**0.011**
Hospital stay, days (median, range)	18 (5 – 74)	19 (6 – 377)	0.354
Intensive Care Unit, days (median, range)	5 (1 – 35)	6 (2 – 52)	0.657
Median overall survival, months (95%-CI)	24 (15.7 - 32.3)	42 (17.1 - 66.9)	**0.003**

Data are presented as n (%), mean +/- SD or median (range). *SD* standard deviation, *PDAC* pancreatic ductal adenocarcinoma, *CAMP* ampullary carcinoma, *DGE* delayed gastric emptying, *PPH* postpancreatectomy hemorrhage, *CR-POPF* clinical relevant pancreatic fistula, *CI* confidence interval. *p*-values < 0.05 are emphasised in bold print

**Table 7:** Cox regression of the unmatched cohort

	Univariable Cox regression			Multivariable Cox regression		
Parameters	HR	95 % CI	*p*-value	HR	95 % CI	*p*-value
CAMP vs. PDAC	0.45	0.35-0.59	<0.001	0.67	0.50-0.89	0.007
PG	0.70	0.58-0.85	0.702			
PJ	1.42	1.18-1.72	<0.001	1.49	1.22-1.82	<0.001
Laparoscopic resection	0.74	0.59-0.93	0.008	0.74	0.59-0.93	0.011
Portal vein resection	0.59	0.49-0.72	<0.001			
T1	0.45	0.0-0.68	<0.001			
T2	0.61	0.48-0.77	<0.001			
T3	1.89	1.52-2.27	<0.001	1.74	1.39-2.18	<0.001
T4	1.06	0.69-1.65	0.788	1.70	1.06-2.73	0.029
R0	0.49	0.39-0.60	<0.001	0.59	0.48-0.74	<0.001
R1	2.04	1.65-2.53	<0.001			

*HR* Hazard ratio, *CI* confidence interval, *CAMP* ampullary carcinoma, *PDAC* pancreatic ductal adenocarcinoma, *PG* pancreatogastrostomy, *PJ* pancreaticojejunostomy

### Comparison of survival between the two decades (2002 – 2011 and 2012 – 2021)

**Fig. 4:**
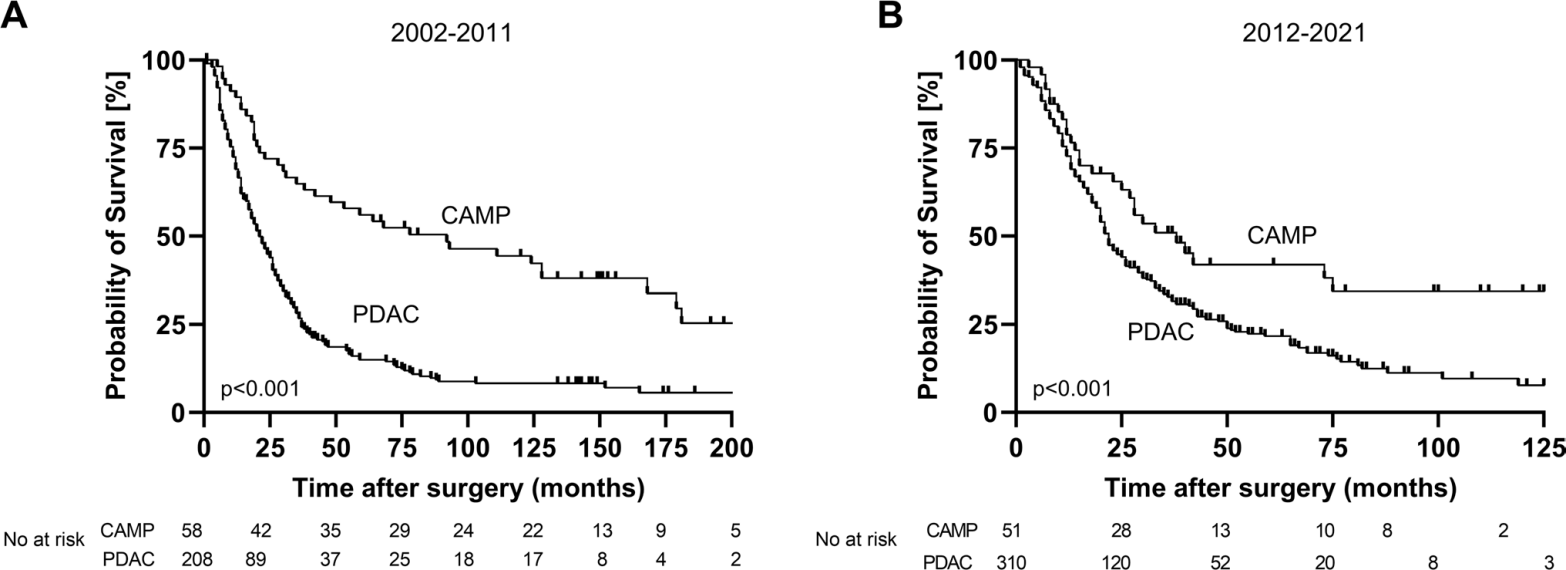
Median overall survival dependent on the decade of surgery.** A**: Overall survival in the first decade (2002-2011). **B**: Overall survival in the second decade (2012 – 2021). PDAC = pancreatic ductal adenocarcinoma. CAMP = ampullary carcinoma

As adiuvant treatment of most malignancies, including PDAC and ampullary carcinomas, has changed over the time, including more aggressive and effective chemotherapy regimens, we divided our patient cohort in two groups, depending on the decade of surgery (2002 – 2011 and 2012 – 2021), in order to evaluate a potential effect of these changes in adiuvant treatment on overall survival of patients. We found a consistently higher rate of adiuvant chemotherapeutical treatment in PDAC patients compared to CAMP patients for both decades: 61.6% PDAC vs. 17.9% CAMP from 2002 – 2011 (*p* < 0.001) and 52.1% PDAC vs. 31.4% CAMP from 2012 – 2021 (*p* = 0.002). In the second decade, we found an increasing adiuvant treatment in the group of CAMP patients compared to the first decade, but the rate of patients with adiuvant treatment remains significantly lower than in the PDAC group. However, in all of our analyses, CAMP patients present with a significantly better overall survival: 92 months (95%-CI 33.4 – 150.6 months) vs. 21 months (95%-CI 17.1 – 24.9 months; *p* < 0.001) from 2002 to 2011 and 33 months (95%-CI 20.3 – 45.7 months) for CAMP patients vs. 21 months (95%-CI 19.1 – 23.0 months) for PDAC patients (*p* = 0.010) from 2012 to 2021. Kaplan Meier curves for both decades are shown in Fig. 4A and B. Details on baseline characteristics and postoperative complications of both decades are summarized in Supplementary Tables 2-7.

## Discussion

Pancreatoduodenectomies are complex surgical procedures associated with a considerable postoperative morbidity and mortality, even in high-volume centers [10–12]. However, so far, they are the only potentially curative treatment for different cancer entities localized in the periampullary region like pancreatic head cancer or ampullary carcinomas [21–23]. In this first analysis of our patient cohort in a high-volume center, we could show that pancreatoduodenectomies seem to differ in performance and complication rate between these two different tumor entities. Whereas pancreatoduodenectomies for PDAC-tumors are characterized by a longer operation time and more venous resections, there are more postoperative surgical complications - especially more clinically relevant pancreatic fistulas - after pancreatoduodenectomies for CAMP-tumors. This higher rate of pancreatic fistulas in CAMP patients may be caused by the typically soft texture of the pancreatic gland in ampullary and distal bile duct tumors and a smaller diameter of the pancreatic duct in these patients [24], which is consistent with our data showing a significantly higher rate of soft pancreatic texture and a smaller main pancreatic duct in our unmatched collective of ampullary carcinoma patients. However, after propensity score matching, this significantly higher rate of CR-POPF in the CAMP group remains consistent, indicating that texture of the pancreatic gland and duct size aren´t the only parameters relevant for this fistula rate. Fortunately, conservative treatment in these patients seems to be quite effective, as the rate of surgical revisions isn´t increased in comparison to PDAC patients. Moreover, postoperative mortality after pancreatoduodenectomies didn´t differ between both groups and lays with 3.7% for CAMP patients in the range of another recent multicenter study analyzing ampullary tumors [25].

In spite of a trend towards more surgery-related complications in pancreatoduodenectomies for ampullary carcinomas, long-term survival rates are particularly favorable in these tumors compared to pancreatic adenocarcinomas. This is comparable to other studies that revealed a better long-term survival in patients with ampullary carcinomas than in PDAC patients or patients with distal bile duct cancer [26, 27]. This improved long-term survival might be caused by a different tumor biology and therefore by the tumor entity itself with ampullary carcinomas representing a less aggressive tumor entity in general in comparison to PDAC tumors. However, there are three subtypes of ampullary carcinomas, namely an intestinal type, a pancreatobiliary type and a mixed type [5, 28], which differ concerning aggressiveness and median overall survival from approximately 115 months for the intestinal subtype down to 16 months in case of a pancreatobiliary type [5]. As data on histopathological subtypes of CAMP patients isn´t provided in our pancreatic surgery database, we scanned the histopathological results from the original patient reports. Unfortunately, data concerning histopathological subtypes was only available for 45 (44.1%) of our ampullary carcinoma patients, so that we aren´t able to give a final statement about the influence of histopathological subtypes on overall survival in our cohort. However, the range of survival time in our CAMP patients from 17 to 69 months might indicate more patients with a pancreatobiliary subtype in our cohort.

By dividing our cohort in two decades, we aimed to analyze a potential effect of changes in chemotherapeutic regimens on the overall survival of our patient cohort. Here, the survival benefit for CAMP patients remains consistent in comparison to PDAC patients, although the rate of patients treated with adiuvant chemotherapy in our CAMP cohort was significantly lower than in the PDAC group.

Another reason for a survival benefit of CAMP patients might be an earlier diagnosis of these tumors due to the early jaundice caused by the location of the tumors. In our collective, ampullary carcinomas presented with a significantly higher rate of T4-tumors compared to PDAC tumors - indicating advanced primary tumors in spite of an expected early diagnosis -, but also with a significantly higher rate of early primary tumors in the T2-stadium. By dividing our cohort in groups of early and advanced primary tumor stages, we could show that the survival benefit of CAMP patients is more distinct in the early primary tumor stages, so that an early diagnosis may contribute to the improved survival of CAMP patients.

This study has some limitations. First, it is a retrospective analysis of our patient cohort. However, due to ampullary carcinomas representing a rare tumor entity, a prospective inclusion of a sufficient number of patients is challenging, so that such a study might be stopped early due to insufficient inclusion of patients. Second, our study is a single center study covering a large period of time, in which there were changes in operative techniques with increasing laparoscopic approaches and different reconstruction techniques as well as improvements in adiuvant treatment of carcinoma patients. In order to overcome these limitations, we performed a propensity score matching on the one hand and a subgroup analysis of the two decades on the other hand. After propensity score matching, we still found a significantly higher rate of clinically relevant pancreatic fistula and a persistent survival benefit in ampullary carcinoma patients. Even in dividing our patient cohort in two decades, we could confirm our results, so that changes in the surgical technique and adiuvant therapy might influence complications and outcome only to a certain degree. In spite of all the changes, the most challenging complication following pancreatoduodenectomies for ampullary carcinomas remains a clinically relevant pancreatic fistula. In the future, different approaches like continuous lavage via drains during the first postoperative days or negative suction techniques in high-risk patients might reduce this complication.

## Conclusion

In conclusion, pancreas head resections remain complex surgical procedures for PDAC as well as for ampullary carcinomas. The better long-term survival in ampullary patients is a consequence of the usually less aggressive tumor biology of this entity. In order to reduce the higher rate of postoperative surgical complications in this cohort of patients, further studies examining promising techniques for the reduction of pancreatic fistulas are warranted.

### Supplementary information

ESM 1 (DOCX 44 kb)
