# Supplementary material for: Pancreatic head resection for carcinoma of the ampulla vateri – better long-term prognosis, but more postoperative complications
Source: Langenbecks Arch Surg. 2024 Apr 17;409(1):129. doi: 10.1007/s00423-024-03319-7 (PMC11024026; doi:10.1007/s00423-024-03319-7)
Supplement: Supplementary file 1 — (DOCX 44 kb) [file 423_2024_3319_MOESM1_ESM.docx]

**Supplementary Material**

**Pancreatic head resection for carcinoma of the ampulla vateri – better long-term prognosis, but more postoperative complications**

Simon Kuesters MD¹^*^, Johanna Sundheimer¹, Uwe A. Wittel MD^1^, Sophia Chikhladze MD^1^, Stefan Fichtner-Feigl MD^1^, Esther A. Biesel MD^1^

^1^ *Department of General- and Visceral Surgery*

*University of Freiburg Medical Center*

*Faculty of Medicine*

*Freiburg, Germany*

**: Current address: Clinic for General-, Visceral- and Vascular Surgery*

*Fürst-Stirum-Klinik*

*Bruchsal, Germany*

Table of contents

[1. Propensity Score Matching 2](#_Toc160791556)

[1.1. Table 1: Propensity Score Matching 2](#_Toc160791557)

[2. Comparison of survival between the two decades 3](#_Toc160791558)

[2.1. Table 2: Demographic and surgical parameters of the first decade (2002-2011) 4](#_Toc160791559)

[2.2. Table 3: Histopathological results oft he first decade (2002-2011) 6](#_Toc160791560)

[2.3. Table 4**:** postoperative complications and overall survival of the first decade (2002-2011) 7](#_Toc160791561)

[2.4. Table 5: Demographic and surgical parameters of the second decade (2012-2021) 8](#_Toc160791562)

[2.5. Table 6: Histopathological results of the second decade (2012-2021) 10](#_Toc160791563)

[2.6. Table 7: postoperative complications and overall survival of the second decade (2012-2021) 11](#_Toc160791564)

## Propensity Score Matching

In our single center analysis, we compare pancreatoduodenectomies for pancreatic ductal adenocarcinoma (PDAC) and for ampullary carcinoma (CAMP) concerning overall survival and postoperative complications as well as 30-day-mortality. As surgical parameters in our cohort are distributed differently between patients with PDAC and patients with CAMP, we performed a propensity score matching to reduce potential biases due to different surgical techniques. A multivariable logistic regression model was performed to generate the propensity score. The following factors were included in this model: laparoscopic resection, reconstruction technique (pancreatogastrostomy vs pancreaticojejunostomy) and portal vein resections. Patients with a soft pancreatic texture were distributed differently as well. As we have only data concerning the pancreatic texture from less than half of our patients, we didn´t include this factor in our propensity score matching. After establishing the propensity score, 1:1 matching using the nearest-neighbour matching was performed with a caliper of 0.01 without replacement. Post hoc balance diagnostic was performed using mean standardized differences. Details concerning propensity score matching are summarized in Supplementary table 1.

## 1.1. Table 1: Propensity Score Matching

|  | **Multivariable** | | |  |
| --- | --- | --- | --- | --- |
| Parameter | **β**^1^ | **OR**^2^ | **95%CI** | **P value** |
| PG vs. PJ | 0.898 | 2.454 | 1.539 – 3.912 | < 0.001 |
| Laparoscopic resection | 0.584 | 1.794 | 1.077 – 2.988 | 0.025 |
| Venous resection | 2.153 | 8.612 | 4.086 – 18.152 | < 0.001 |
|  |  |  |  |  |

PG = pancreatogastrostomy, PJ = pancreaticojejunostomy

## Comparison of survival between the two decades

As not only surgical techniques, but also adjuvant treatment of most malignancies, including PDAC and CAMP, has changed over the time, we divided our patient cohort in two groups depending on the decade of surgery (2002 - 2011 and 2012 – 2021) in order to evaluate a potential effect on overall survival, mortality and complications. After dividing our patient cohort in these two decades, we found similar results concerning overall survival between both decades with a significantly better median survival of ampullary carcinoma patients: 92 months vs. 21 months (95% CI 33.4 – 150.6 months, p < 0.001) in the first decade and 33 months for CAMP patients vs. 21 months for PDAC patients (95% CI 17.1 – 24.9 months, p = 0.010) in the second decade. We didn´t find any differences concerning postoperative complications like delayed gastric emptying (DGE), postpancreatectomy hemorrhage (PPH), wound infections or postoperative complications in general; however, the significantly higher rate of clinically relevant pancreatic fistula remains consistent over both decades. Details concerning baseline characteristics, surgical parameters and histopathological results as well as postoperative complications, mortality and long-term overall survival are summarized in supplementary tables 2 – 7.

## 2.1. Table 2: Demographic and surgical parameters of the first decade (2002-2011)

|  | **PDAC**  **(n = 208)** | **CAMP**  **(n = 58)** | ***p* value** |
| --- | --- | --- | --- |
| **Demographic parameters and comorbidities** | | | |
| Age, years | 65.5 (10.6) | 65.8 (10.8) | 0.839 |
| Sex   - male - female | 98 (47.1)  110 (52.9) | 33 (56.9)  25 (43.1) | 0.188 |
| BMI, kg/m2 | 24.6 (3.8) | 25.3 (3.7) | 0.214 |
| ASA classification |  | | |
| - ASA 1 | 13 (6.3) | 4 (6.9) | 0.859 |
| - ASA 2 | 129 (62.0) | 34 (20.9) | 0.638 |
| - ASA 3 | 64 (30.8) | 20 (34.5) | 0.591 |
| - ASA 4 | 2 (1.0) | 0 (0.0) | 0.453 |
| Comorbidities (n = 153) | 95 (76.6) | 25 (86.2) | 0.258 |
| Coronary heart disease (n = 148) | 19 (15.8) | 5 (17.9) | 0.794 |
| Hypertension (n = 150) | 64 (52.5) | 18 (64.3) | 0.257 |
| Lung disease (n = 153) | 16 (12.9) | 5 (17.2) | 0.541 |
| Renal disease (n = 165) | 11 (8.3) | 2 (6.1) | 0.665 |
| Liver disease (n = 143) | 52 (44.4) | 11 (42.3) | 0.843 |
| Diabetes mellitus | 49 (23.7) | 11 (19.0) | 0.449 |
| Alcohol abuse | 16 (9.3) | 4 (8.0) | 0.777 |
| Nicotin abuse | 28 (16.4) | 11 (22.0) | 0.359 |
| Preoperative icterus | 139 (67.5) | 38 (65.5) | 0.779 |
| Preoperative bile duct stent | 130 (62.8) | 31 (53.4) | 0.197 |
| neoadiuvant chemotherapy (n = 252) | 13 (6.3) | 0 (0.0) | **0.089** |
| Adiuvant therapy (n = 185) | 90 (61.6) | 7 (17.9) | **< 0.001** |
| **Surgical parameters** | | | |
| Duration of surgery, minutes | 438 (91.8) | 410 (79.4) | **0.037** |
| Venous resection | 93 (44.7) | 4 (7.0) | **< 0.001** |
| Soft pancreas | 36 (25.5) | 20 (48.8) | **0.002** |
| Laparoscopical-assisted resection | 6 (2.9) | 4 (6.9) | 0.155 |
| Reconstruction technique |  |  |  |
| - pancreatogastrostomy | 115 (55.3) | 42 (72.4) | **0.019** |
| - pancreaticojejunostomy | 93 (44.7) | 16 (27.6) | **0.019** |

Data are presented as n (%), or mean +/- SD. SD = standard deviation. PDAC = pancreatic ductal adenocarcinoma. CAMP = ampullary carcinoma. BMI = body mass index. ASA = American Society of Anesthesiologists.

## 2.2. Table 3: Histopathological results oft he first decade (2002-2011)

|  | **PDAC**  **(n = 208)** | **CAMP**  **(n = 58)** | ***p* value** |
| --- | --- | --- | --- |
| Grading |  | | |
| - G1 | 6 (2.9) | 4 (7.1) | **0.148** |
| - G2 | 127 (62.3) | 36 (64.3) | 0.781 |
| - G3 | 67 (32.8) | 16 (28.6) | 0.544 |
| - G4 | 4 (2.0) | 0 (0.0) | 0.291 |
| Resection margin |  | | |
| - R0 | 152 (73.1) | 56 (96.6) | **< 0.001** |
| - R1 | 54 (26.0) | 2 (3.4) | **< 0.001** |
| - R2 | 2 (1.0) | 0 (0.0) | 0.453 |
| TNM classification |  | | |
| - T1 | 8 (3.9) | 10 (17.2) | **< 0.001** |
| - T2 | 17 (8.3) | 23 (39.7) | **< 0.001** |
| - T3 | 171 (83.0) | 18 (31.0) | **< 0.001** |
| - T4 | 10 (4.9) | 7 (12.1) | **0.048** |
| - N0 | 60 (29.1) | 27 (46.6) | **0.013** |
| - N1 | 145 (70.4) | 31 (53.4) | **0.016** |
| - N2 | 1 (0.5) | 0 (0.0) | 0.595 |

Data are presented as n (%). PDAC = pancreatic ductal adenocarcinoma. CAMP = ampullary carcinoma

## 2.3. Table 4**:** postoperative complications and overall survival of the first decade (2002-2011)

|  | **PDAC**  **(n = 208)** | **CAMP**  **(n = 58)** | ***p* value** |
| --- | --- | --- | --- |
| DGE B/C | 45 (21.6) | 17 (29.3) | 0.221 |
| PPH B/C | 15 (7.2) | 5 (8.6) | 0.719 |
| CR-POPF | 16 (7.7) | 17 (29.3) | **< 0.001** |
| Blood transfusion | 52 (25.0) | 9 (15.5) | 0.129 |
| Wound infection | 31 (14.9) | 14 (25.0) | 0.075 |
| Urinary tract infection | 18 (8.7) | 2 (3.5) | 0.193 |
| Thrombembolism | 2 (1.0) | 1 (1.8) | 0.606 |
| Intraabdominal abscess | 16 (7.7) | 8 (14.0) | 0.139 |
| Pneumonia | 5 (2.4) | 2 (3.5) | 0.645 |
| Reintubation | 9 (4.3) | 1 (1.8) | 0.377 |
| Sepsis | 7 (3.4) | 1 (1.8) | 0.529 |
| Acute kidney failure | 4 (1.9) | 0 (0.0) | 0.296 |
| Insufficiency pancreaticojejunostomy | 10 (4.8) | 2 (3.4) | 0.659 |
| Insufficiency pancreatogastrostomy | 8 (3.8) | 7 (12.1) | **0.016** |
| Insufficiency biliodigestive anastomosis | 2 (1.0) | 1 (1.7) | 0.630 |
| Postoperative mortality | 2 (1.0) | 0 (0.0) | 0.452 |
| Any complication | 120 (57.7) | 37 (63.8) | 0.403 |
| Surgery-related complication | 79 (38.0) | 33 (58.9) | **0.005** |
| Surgical revision | 20 (9.6) | 8 (13.8) | 0.359 |
| Postoperative interventional therapy | 55 (26.6) | 21 (36.8) | 0.129 |
| Postoperative conservative therapy | 112 (55.7) | 41 (75.9) | **0.007** |
| Hospital stay in days | 19 (6 - 329) | 22.5 (6 – 318) | 0.081 |
| Intensive Care Unit in days | 5 (2 - 29) | 6 (3 – 52) | **0.024** |
| Median overall survival, months (95% CI) | 21 (17.1 – 24.9) | 92 (33.4 – 150.6) | **< 0.001** |

Data are presented as n (%), mean +/- SD or median (range). SD = standard deviation. PDAC = pancreatic ductal adenocarcinoma. CAMP = ampullary carcinoma. DGE = delayed gastric emptying. PPH = postpancreatectomy hemorrhage. CR-POPF = clinical relevant pancreatic fistula. CI = confidence interval.

## 2.4. Table 5: Demographic and surgical parameters of the second decade (2012-2021)

|  | **PDAC**  **(n = 310)** | **CAMP**  **(n = 51)** | ***p* value** |
| --- | --- | --- | --- |
| **Demographic parameters and comorbidities** | | | |
| Age, years | 66.8 (11.5) | 67.3 (12.0) | 0.783 |
| Sex   - male - female | 171 (55.2)  139 (44.8) | 26 (51.0)  25 (49.0) | 0.578 |
| BMI, kg/m2 | 25.4 (4.9) | 24.6 (3.8) | 0.237 |
| ASA classification |  | | |
| - ASA 1 | 9 (2.9) | 0 (0.0) | 0.218 |
| - ASA 2 | 120 (38.7) | 23 (45.1) | 0.387 |
| - ASA 3 | 170 (54.8) | 27 (52.9) | 0.801 |
| - ASA 4 | 11 (3.5) | 1 (2.0) | 0.558 |
| Comorbidities | 248 (80.0) | 40 (80.0) | 1.000 |
| Coronary heart disease | 42 (13.5) | 6 (12.0) | 0.765 |
| Hypertension | 171 (55.2) | 27 (54.0) | 0.878 |
| Lung disease | 57 (18.4) | 12 (24.0) | 0.349 |
| Renal disease | 32 (10.3) | 5 (10.0) | 0.944 |
| Liver disease | 70 (22.6) | 7 (14.0) | 0.170 |
| Diabetes mellitus | 76 (24.5) | 6 (12.0) | 0.050 |
| Alcohol abuse | 31 (10.0) | 6 (12.0) | 0.666 |
| Nicotin abuse | 69 (22.3) | 12 (24.0) | 0.784 |
| Preoperative icterus | 204 (66.7) | 32 (65.3) | 0.851 |
| Preoperative bile duct stent | 153 (50.2) | 37 (75.5) | **< 0.001** |
| neoadiuvant chemotherapy | 27 (8.7) | 0 (0.0) | **0.028** |
| Adiuvant therapy (n = 358) | 160 (52.1) | 16 (31.4) | **0.002** |
| **Surgical parameters** | | | |
| Duration of surgery, minutes | 428 (97.6) | 370 (89.8) | **<0.001** |
| Venous resection | 126 (40.6) | 4 (7.8) | **< 0.001** |
| Soft pancreas (n = 199) | 60 (35.1) | 16 (57.1) | 0.084 |
| Laparoscopical-assisted resection | 106 (34.2) | 29 (56.9) | **0.002** |
| Reconstruction technique |  |  |  |
| - pancreatogastrostomy | 40 (12.9) | 11 (21.6) | 0.100 |
| - pancreaticojejunostomy | 270 (87.1) | 40 (78.4) | 0.100 |
| Diameter of pancreatic main duct, mm (n = 118) | 5.1 (2.2) | 3.8 (1.7) | **0.038** |

Data are presented as n (%), or mean +/- SD. SD = standard deviation. PDAC = pancreatic ductal adenocarcinoma. CAMP = ampullary carcinoma. BMI = body mass index. ASA = American Society of Anesthesiologists.

## 2.5. Table 6: Histopathological results of the second decade (2012-2021)

|  | **PDAC**  **(n = 310)** | **CAMP**  **(n = 51)** | ***p* value** |
| --- | --- | --- | --- |
| Grading |  | | |
| - G1 | 8 (2.7) | 4 (8.2) | 0.057 |
| - G2 | 154 (52.9) | 25 (51.0) | 0.805 |
| - G3 | 127 (43.6) | 20 (40.8) | 0.712 |
| - G4 | 2 (0.7) | 0 (0.0) | 0.561 |
| Resection margin |  | | |
| - R0 | 240 (77.7) | 50 (98.0) | **< 0.001** |
| - R1 | 63 (20.4) | 1 (2.0) | **0.001** |
| - R2 | 6 (1.9) | 0 (0.0) | 0.316 |
| TNM classification |  | | |
| - T1 | 24 (7.8) | 5 (10.0) | 0.591 |
| - T2 | 87 (28.2) | 14 (28.0) | 0.982 |
| - T3 | 196 (63.4) | 25 (50.0) | 0.070 |
| - T4 | 2 (0.6) | 6 (12.0) | **< 0.001** |
| - N0 | 91 (29.4) | 24 (47.1) | **0.012** |
| - N1 | 170 (55.0) | 22 (43.1) | 0.115 |
| - N2 | 48 (15.5) | 5 (9.8) | 0.285 |

Data are presented as n (%). PDAC = pancreatic ductal adenocarcinoma. CAMP = ampullary carcinoma

## 2.6. Table 7: postoperative complications and overall survival of the second decade (2012-2021)

|  | **PDAC**  **(n = 310)** | **CAMP**  **(n = 51)** | ***p* value** |
| --- | --- | --- | --- |
| DGE B/C | 72 (23.6) | 11 (22.4) | 0.859 |
| PPH B/C | 22 (9.8) | 1 (3.0) | 0.202 |
| CR-POPF | 48 (15.6) | 16 (32.0) | **0.005** |
| Blood transfusion | 45 (14.6) | 3 (5.9) | 0.091 |
| Wound infection | 36 (11.7) | 7 (13.7) | 0.672 |
| Urinary tract infection | 15 (4.9) | 0 (0.0) | 0.108 |
| Thrombembolism | 12 (3.9) | 2 (3.9) | 0.990 |
| Intraabdominal abscess | 39 (12.6) | 5 (9.8) | 0.569 |
| Pneumonia | 16 (5.2) | 1 (2.0) | 0.316 |
| Reintubation | 13 (4.2) | 3 (5.9) | 0.591 |
| Sepsis | 14 (4.5) | 1 (2.0) | 0.395 |
| Acute kidney failure | 17 (5.5) | 0 (0.0) | 0.086 |
| Insufficiency pancreaticojejunostomy | 11 (3.6) | 2 (3.9) | 0.898 |
| Insufficiency pancreatogastrostomy | 4 (1.3) | 0 (0.0) | 0.414 |
| Insufficiency biliodigestive anastomosis | 9 (2.9) | 0 (0.0) | 0.217 |
| Postoperative mortality | 19 (6.1) | 4 (7.8) | 0.642 |
| Any complication | 170 (55.0) | 33 (64.7) | 0.196 |
| Surgery-related complication | 133 (42.9) | 29 (56.9) | 0.063 |
| Surgical revision | 46 (14.9) | 5 (9.8) | 0.335 |
| Postoperative interventional therapy | 77 (24.9) | 12 (23.5) | 0.831 |
| Postoperative conservative therapy | 187 (60.5) | 35 (70.0) | 0.200 |
| Hospital stay in days | 16 (2 – 120) | 17 (6 – 377) | 0.132 |
| Intensive Care Unit in days | 5 (1 – 68) | 4 (2 -29) | 0.192 |
| Median overall survival, months (95% CI) | 21 (19.1 – 23.0) | 33 (20.3 – 45.7) | **0.010** |

Data are presented as n (%), mean +/- SD or median (range). SD = standard deviation. PDAC = pancreatic ductal adenocarcinoma. CAMP = ampullary carcinoma. DGE = delayed gastric emptying. PPH = postpancreatectomy hemorrhage. CR-POPF = clinical relevant pancreatic fistula. CI = confidence interval.
